# Relation between voxel-based specific regional analysis system for Alzheimer's disease (VSRAD) on 3-tesla MRI and cognitive performances: Practical application in clinical settings

**DOI:** 10.1016/j.ibneur.2026.06.018

**Published:** 2026-07-06

**Authors:** K. Igase, M. Igase, H. Matsuda, J. Hara, J.R. Bock, K. Sadamoto, W.R. Shankle

**Affiliations:** a Department of Advanced Neurosurgery, Ehime University Graduate School of Medicine, Japan; b Department of Neurosurgery, Washokai Sadamoto Hospital, Japan; c Department of Antiaging Medicine, Ehime University Graduate School of Medicine, Japan; d Integrative Brain Imaging Center, National Center of Neurology and Psychiatry, Tokyo, Japan; e Medical Care Corporation, Newport Beach, CA, USA; f Shankle Clinic, Newport Beach, CA, USA; g Pickup Family Neurosciences Institute, Hoag Memorial Hospital Presbyterian, Newport Beach, CA, USA

**Keywords:** VSRAD, Cognitive Impairment, Mild Cognitive Impairment, MCI screen, 3Tesla MRI

## Abstract

**Background:**

Voxel-based specific regional analysis system for Alzheimer's disease (**VSRAD**) software using MRI scanner allows quantification of hippocampal and parahippocampal atrophy in the medial temporal structures by Z-score, and this score is widely used in clinical Alzheimer's disease (**AD**) diagnosis. However, it is unclear whether the Z-score is useful to discriminate normal aging from cognitive impairment (**CI**) or mild cognitive impairment (**MCI**). The present study examined the associations between VSRAD Z-score and cognitive performance quantified by Memory Performance Index (**MPI**) and determined a Z-score cut-off value.

**Method:**

Three-tesla brain MRI was conducted in 100 outpatients without dementia, and all MRI data were analyzed using VSRAD. The target region of interest (**ROI**) mainly consisted of the para hippocampal gyrus. The degree of atrophy in the ROI was obtained from the averaged positive Z-score of the ROI. Cognitive performance was evaluated with the Japanese version of the MCI screen (**MCIS**). Patients were classified into normal (**NL**) and below normal (**BNL**) cognitive groups by MPI. The relation between MPI and VSRAD Z-score were assessed with logistic regression analysis, and the cut-off value for Z-score was determined by receiver operating characteristic curve analysis.

**Results:**

Sixty-two percent (62%) were identified as the BNL group by MPI. Univariate analyses found that the BNL group had a significantly higher age, shorter years of education, and higher Z-score in VSRAD compared to the NL group, but no statistically significant difference was observed between genders. Bivariate correlation found that MPI, which is adjusted for age, gender, and years of education, was significantly correlated with Z-score assessed by VSRAD (Pearson’s *r* = -0.52, *p* < .001). A subsequent logistic regression of VSRAD Z-score on BNL classification was used to generate a receiver operating characteristic curve (AUC = 0.75). The Youden index was applied to identify a cut-off value of VSRAD Z-score of 1.14 (sensitivity = 62.9%; specificity = 84.2%) to classify MPI < 50.2 (BNL) with overall accuracy of 73.5%.

**Conclusions:**

VSRAD Z-score using VSRAD software was one independent factor significantly associated with cognitive performance measured by MPI. The determination of a cut-off value for Z-score (1.14) that can help discriminate normal patients from those with MCI.

## Introduction

With the rapid aging of our population, cognitive impairment (**CI**) leads to healthcare and socioeconomic burdens to our society and also negatively impacts quality of life for those with CI and their families. There are many conditions that cause CI including neurodegenerative disorders, such as Alzheimer’s disease (**AD**), vascular dementia, and Lewy body dementia, as well as other more treatable conditions, such as depression, heart and cardiovascular disease, and diabetes. While both research and clinical communities agree that prevention and early detection of underlying conditions for CI play a key role in improving care outcomes, and that recent clinical practice recommendations for mild cognitive impairment (**MCI**) emphasize the importance of objectively identifying MCI and managing it (Langa and Levine, 2014, Petersen et al., 2018), a practical and objective way of doing so in clinical settings is yet to be established.

There are two such objective tools that could be easily integrated into clinical settings: the MCI Screen (**MCIS**) and the voxel-based specific regional analysis system for Alzheimer's disease (**VSRAD**). The MCIS is a 10-minute, computationally scored, and staff-administered cognitive test. It is highly accurate in distinguishing MCI and mild dementia (97% and 99% accurate, respectively) from normal aging (Shankle et al., 2005). The MCIS generates a Memory Performance Index (**MPI**) (Shankle et al., 2009), a quantitative measure of the respondent’s pattern of recall on a 0–100 scale, and it classifies the score based on norms for the respondent’s demographic peers. The VSRAD is a magnetic resonance imaging (**MRI**)-based volumetric software that quantifies the regional brain atrophy as a Z-score, specifically in the medial temporal structures, including the hippocampus and entorhinal cortex (Goto et al., 2008). Besides, it has been reported that not only AD, but also cardiovascular diseases (Nishimura et al., 2007) and diabetes (Hirabayashi et al., 2016) that cause CI can lead to hippocampal atrophy, making VSRAD analysis potentially useful.

While VSRAD Z-score has been widely used for AD diagnostic evaluation using 1.5-tesla MRI scans (VSRAD−1.5 T), its utilities with 3-tesla MRI scans (VSRAD-3T) have also been studied (Sone et al., 2018). However, its accuracy for discriminating MCI from normal subjects has not been well-studied. Therefore, in this study, we will 1) examine the relationship between MCIS’s MPI score and VSRAD’s Z-score with VSRAD-3T, and 2) determine the optimal cut-off VSRAD Z-score for MCI.

## Methods

### Study subjects

The initial study participants were 108 consecutive patients who visited Washokai Sadamoto Hospital in Ehime Prefecture with complaints of subjective memory impairment between May 2017 and March 2022. All participants underwent the Mini-Mental State Examination (MMSE), where one patient who was unable to execute the MMSE and seven patients with the MMSE score of less than 24 were excluded. The analysis was performed on 100 patients, of whom 64 (64%) were female, between 47 and 90 years old (M = 75.90; SD = 7.56). All subjects were of Japanese origin and signed written informed consent approved by the institutional ethical committees of Washokai Sadamoto hospital. Written informed consent was obtained from all participants.

### MCI screen

For this study, the MCIS was selected for its high sensitivity and specificity for identifying mild cognitive impairment (**MCI**) (95% and 88%, respectively) (Shankle et al., 2005), cross-validation with other assessment tests (Rafii et al., 2011), much higher accuracy (97% for MCI) over commonly used assessment tools in primary care settings (e.g., the Mini-Mental State Exam and Clock Drawing Test) (Trenkle et al., 2007), and its utility and adoptability in clinical settings.

All subjects were assessed by the Japanese version of the MCIS (Cho et al., 2008), and MPI was reported. The MCIS consists of multi-trial free immediate and delayed recall of a list of 10 semantically-controlled words (Shankle et al., 2005). During the MCIS assessment, the respondents are verbally presented the 10 words across three learning trials with the word presentation order held constant for each trial. The respondents are then asked to recall as many words as possible, in any order, after each presentation. After an unrelated cognitive task lasting approximately 5 min, respondents are asked again to recall as many words as possible. Immediately after the test administration, the MPI score gets generated, quantifying overall memory performance and mapping the MPI on a 0–100 scale. The MPI calculated from MCIS is separated by borderline value 50.2, that is, the value below 50.2 diagnosed as MCI, whereas the value over 50.2 as normal. In this study the MPI is adjusted for age, gender, and years of education. Based on the MPI, subjects were classified into two groups: *below normal* (**BNL**: MPI <50.2) and *normal* (**NL**: MPI ≥ 50.2) (Shankle et al., 2009), where subjects in former group were suspected of MCI and those in latter group are deemed to be almost healthy aging with some memory complaints.

### VSRAD score acquisition

All subjects underwent MRI with a 3-tesla scanner (Signa Excite 3.0 T; GE Healthcare, Milwaukee, WI) for this study. VSRAD image was acquired with following parameters: slice orientation = sagittal; repetition time/echo time = 7.2/3.4 msec; scan time = 144 s; section thickness = 1.2 mm; flip angle = 10°; field of view = 240 × 180; matrix = 256 × 256; and voxel volume = 1.32 mm^3^. VSRAD’s Z-score was evaluated using acquired imaging data using VSRAD (VSRAD® advance 2, Eisai, Japan) software that quantitatively calculates the extent of brain atrophy (percent of volume reduction in gray and white matter) compared to an MRI imaging database of 80 age-matched healthy controls, based on voxel-based morphometry (VBM) (Matsuda, 2016). Z-score [(patient’s voxel-level – normal control average of voxel-level) / (normal control standard deviation)] was calculated in each voxel and assessed percentage of temporal gray matter atrophy in all patients.

### Statistical analysis

All continuous and categorical variables are presented in Table 1 as means (***M***) and standard deviations (***SD***) or count and percent, respectively, for the overall sample and separately for BNL and NL groups. Univariate comparisons between the two groups were assessed using independent *t*-tests for continuous variables and χ^2^ tests for categorical for variables. A multivariate logistic regression analysis was performed, using backward elimination with likelihood-ratio test statistics to sequentially remove non-significant predictors from the model until a reduced model was identified, where covariates (age, gender, and education) were included. The relation between MPI and VSRAD Z-score was clarified with logistic regression analysis, and since the MPI and VSRAD Z-scores are two paired variables and both follow a normal distribution, we used Pearson's product-moment correlation coefficient, a parametric method. The cut-off value for VSRAD Z-score (NL vs. BNL) was determined by receiver operating characteristic curve analysis. In all comparisons, a two-tailed p < 0.05 was considered statistically significant. Analyses were performed using the Stata software package for Windows version 15.1.

**Table 1:** Summary statistics, univariate analyses, and multivariate logistic regression.

	**BNL**	**NL**	**Total**	**Univariate**^a^	**Logistic Model**^b^
	***n*****= 62**	***n*****= 38**	***n*****= 100**	***t*****/ χ**^**2**^	***OR*****(95% CI)**
**Age in yrs.,*****M*** **(*****SD*****)**	**79.50 (4.69)**	**70.03 (7.71)**	**75.90 (7.56)**	**7.65 ‡**	**1.35** **(1.17; 1.56) ‡**
**Female, count** **(%)**	**37** **(60%)**	**27** **(71%)**	**64** **(64%)**	**1.32**	
**Education in yrs.,*****M*****(*****SD*****)**	**11.18 (1.78)**	**12.13 (1.80)**	**11.54 (1.84)**	**−2.59 †**	
**MPI,*****M*** **(*****SD*****)**	**36.04 (9.37)**	**61.52 (6.89)**	**45.72 (15.05)**	**−14.52 ‡**	
**MMSE,*****M*** **(SD)**	**25.63** **(2.18)**	**28.71** **(1.94)**	**27.27** **(1.95)**	**−11.53 ‡**	
**Z-score,*****M*** **(*****SD*****)**	**1.59 (0.97)**	**0.86 (0.35)**	**1.31 (0.87)**	**4.47 ‡**	**3.47** **(1.29; 9.37) †**

Note. BNL = Below normal cognition; NL = normal cognition; OR = Odds ratio; CI = confidence interval; MPI = memory performance index; MMSE: Mini-Mental State Examination.

*p < 0.05; † p < 0.01; ‡ p < 0.001.

a Independent *t*-tests were performed for continuous variables; a χ2 test was performed for categorical gender.

b Multivariate logistic regression variables were sequentially removed using backward elimination: gender (p = 0.827), education (p = 0.597).

## Results

Of 100 subjects, 62 (62%) were classified via MPI score as BNL. Table 1 summarizes subject characteristics. Univariate analyses found that the BNL group had a significantly higher age, shorter years of education, and higher Z-score in VSRAD compared to the NL group, but no statistically significant difference was observed between genders. The multivariate logistic regression model, after backward elimination, included age (OR = 1.35 [95% CI = 1.17; 1.56], *p* < 0.001) and VSRAD Z-score (OR = 3.47 [95% CI = 1.29; 9.37], *p* = 0.01).

**Fig. 1:**
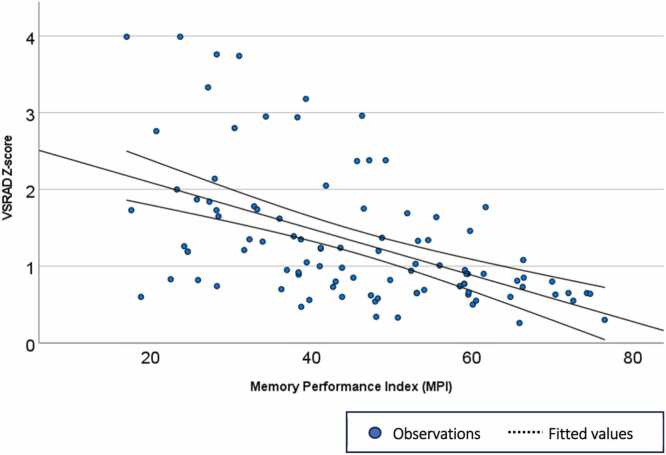
Correlation between MPI and Z-score. MPI was significantly correlated with Z-score assessed by VSRAD (Pearson’s *r* = −0.52, *p* < 0.001). Straight line reveals the regression line and upper and lower curve lines indicate the confidence interval. Note: MPI = memory performance index; VSRAD = Voxel-based specific regional analysis system.

**Fig. 2:**
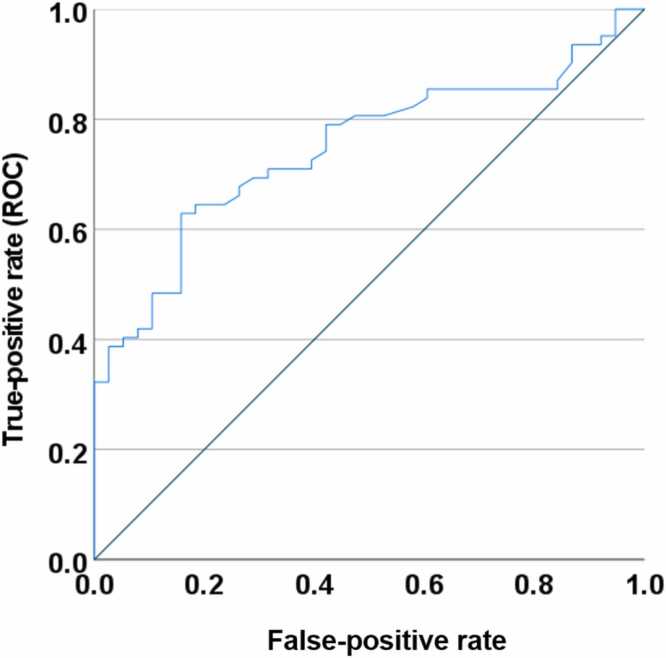
ROC curve analysis for the presence of MCI. AUC = 0.75. A cutoff score was generated with the Youden index for maximization of sensitivity and specificity: VSRAD Z-score = 1.14; sensitivity = 62.9%; specificity = 84.2%; accuracy = 73.5%. Note: ROC = receiver operating characteristic; MCI = mild cognitive impairment; AUC: area under the curve.

Bivariate correlation found that MPI was significantly correlated with Z-score assessed by VSRAD (Pearson’s *r* = −0.52, *p* < 0.001; Fig. 1). A subsequent logistic regression of VSRAD Z-score on BNL classification was used to generate a receiver operating characteristic curve (AUC = 0.75; Fig. 2). The Youden index was applied and obtained as *J*= 0.47 to identify a cut-off value of VSRAD Z-score of 1.14 (sensitivity = 62.9%; specificity = 84.2%) to classify MPI < 50.2 (BNL) with overall accuracy of 73.5%.

## Discussion

The result demonstrated a significant correlation between the VSRAD Z-score and the MPI score, suggesting a utility of VSRAD Z-score for early detection of MCI in clinical settings. The result also identified an optimal cut-off value of VSRAD Z-score (1.14) for distinguishing BNL group (subjects with MCI) from NL group (subjects with normal cognition). The previous study (Oshikubo et al., 2020), which was performed for diagnosing AD, has identified a cut-off score of 1.57 for mean value of positive Z-scores in the target volume-of-interest with 69.4% accuracy in discriminating patients with AD. This study supports our findings of the cut-off score of 1.14, 0.43 points lower than for those for AD, for distinguishing NL group from those with MCI. However, applied Youden index in our study was 0.47, implying that cut-off value of VSRAD Z-score (1.14) may not be appropriate, where the difference of diagnostic criteria between AD and MCI would be involved. In other words, while AD has clear diagnostic criteria, the criteria for MCI are slightly ambiguous, which may be the cause of the discrepancy. Therefore, at present, it is advisable to use our cutoff score of 1.14 in VSRAD z-score as supplementary information.

To date, there are few studies utilizing 3-tesla MRI scan (Niida et al., 2011, Shimoda et al., 2015, Sugiyama et al., 2017) for the purpose of diagnosis in the field of neurology. The current study using VSRAD software based on the 3-tesla MRI scan will further validate the utility of 3-tesla MRI and VSRAD software in clinical settings.

With rapidly increasing needs for prevention and early detection of MCI, the combined use of MPI and VSRAD Z score should be considered. However, further validation studies using larger cohorts are inevitable.

Several limitations of this study are noteworthy. First, MCI screen we used have some findings (Trenkle et al., 2007, Cho et al., 2008), however it is not widely used in the world, whereas the Montreal Cognitive Assessment (MoCA) test is reported to better meet the criteria for screening for detection of MCI among patients aged over 60 years (Ciesielska et al., 2016). If we had also used the MoCA test, the accuracy of our results may have improved. Basically, MoCA has higher sensitivity to executive dysfunction, which is common in early vascular MCI, meanwhile MCIS is heavily focused on episodic memory. Using MoCA test in conjunction with MCIS may have led another cutoff value in VSRAD Z-score, that should be verified in the future. Second, regarding MCI assessment, our analyses were retrospective, and a degree of selection bias may have been introduced. Study participants were recruited from outpatients in our hospital, and accordingly the participants may not necessarily have represented the general population.

## Ethics Approval and Consent to Participate

This study was reviewed and approved by the institutional ethical committees of Washokai Sadamoto Hospital (Approval No. 42). Written informed consent was obtained from all individual participants included in the study.

## Declaration of Competing Interest

None.
